# A comprehensive comparison of medication strategies for platinum-sensitive recurrent ovarian cancer: A Bayesian network meta-analysis

**DOI:** 10.3389/fphar.2022.1010626

**Published:** 2022-11-10

**Authors:** Yuanzhi Liu, Yilan Huang, Jingyan Li, Shengli Wan, Nan Jiang, Jie Yang, Sawitree Chiampanichayakul, Singkome Tima, Songyot Anuchapreeda, Jianming Wu

**Affiliations:** ^1^ Department of Pharmacy, The Affiliated Hospital of Southwest Medical University, Luzhou, Sichuan, China; ^2^ Department of Medical Technology, Faculty of Associated Medical Sciences, Chiang Mai University, Chiang Mai, Thailand; ^3^ School of Pharmacy, Southwest Medical University, Luzhou, Sichuan, China; ^4^ Department of Cardiovascular Surgery, Affiliated Hospital of Southwest Medical University, Luzhou, Sichuan, China; ^5^ Center for Research and Development of Natural Products for Health, Chiang Mai University, Chiang Mai, Thailand; ^6^ Key Laboratory of Medical Electrophysiology of Ministry of Education of China, Medical Key Laboratory for Drug Discovery and Druggability Evaluation of Sichuan Province, Luzhou Key Laboratory of Activity Screening and Druggability Evaluation for Chinese Materia Medica, Southwest Medical University, Luzhou, Sichuan, China

**Keywords:** medication strategies for platinum-sensitive recurrent ovarian cancer recurrent ovarian, platinum-sensitive, chemotherapy, initial therapy, maintenance therapy, network meta-analysis

## Abstract

**Background:** The Platinum-based combination has been proven to have an outstanding effect on patients with platinum-sensitive recurrent ovarian cancer (PSROC), but the best scientific combination has not been established yet. The present study is aimed to seek the best treatment plan for PSROC.

**Methods:** We did a systematic review and Bayesian network meta-analysis, during which lite before March 2022 were retrieved on PubMed, Embase, Web of Science, and Cochrane Central Registry of Controlled databases. We included randomized controlled clinical trials comparing chemotherapy combinations with other treatments for patients with PSROC. The important outcomes concerned were progression-free survival (PFS) (the primary outcome), overall survival (OS), objective response rate (ORR), adverse events (AEs), and AEs-related discontinuation. All outcomes were ranked according to the surface under the cumulative ranking curve.

**Results:** 26 trials involving 10441 patients were retrieved in this study. For the initial treatment of PSROC, carboplatin plus pegylated liposomal doxorubicin (PLD) plus bevacizumab had the best PFS [hazard ratio (HR) 0.59, 95% credible interval (CI) 0.51–0.68]; Carboplatin plus paclitaxel plus bevacizumab resulted in the best OS (HR 1.22, 95% CI 1.09–1.35) and ORR [odds ratio (OR) 1.22, 95% CI 1.09–1.35]. For the maintenance therapy in PSROC, poly (ADP-ribose) polymerase inhibitors (PARPi) following platinum-based chemotherapy provided the best PFS (HR 0.64, 95% CI 0.61–0.68), the highest frequency of adverse events of grade three or higher (OR 0.18, 95% CI 0.07–0.44) but the treatment discontinuation was generally low. Subgroup analysis suggested that trabectedin plus PLD was comparable to single platinum in prolonging PFS in the platinum-free interval (6–12 months).

**Conclusion:** Both platinum-based chemotherapy plus PARPi and platinum-based chemotherapy plus bevacizumab had higher survival benefits than other treatments in PSROC. Trabectedin plus PLD might be a potential alternative treatment strategy for the partially platinum-sensitive subpopulation with intolerance to platinum.

**Systematic Review Registration**: [https://www.crd.york.ac.uk/prospero/display_record.php?], identifier [CRD42022326573].

## 1 Introduction

As one gynecological cancer, ovarian cancer is the fifth leading cause of death for women. At the time of diagnosis, approximately 80% of women have been at the advanced stage, and even after the first-session therapy, around 75% of them also observed a recurrence of ovarian cancer (Arend et al., 2020). Recurrent ovarian cancer (ROC) is divided into three categories. When the platinum-free interval (PFI) is over 6 months after the last dose of platinum, patients with recurrent ovarian cancer are defined as potentially platinum-sensitive and are more likely to achieve a response to further platinum-based chemotherapy (Markman et al., 1991). The other two patterns, the potentially platinum-resistant (PFI from 1 to 6 months), and the platinum-refractory (progressing through platinum or PFI <1 month), adopt various therapies except for platinum agents (Bouberhan et al., 2019).

Currently, according to the latest guidelines from the National Comprehensive Cancer Network (Armstrong et al., 2021), and the International Federation of Gynecology and Obstetrics (Berek et al., 2021), platinum-based treatment strategies are typically proposed for platinum-sensitive, recurrent ovarian cancer (PSROC). The treatment strategies are involved in various classes of drugs, including single-agent platinum (i.e., carboplatin or cisplatin) or paclitaxel for patients who failed to tolerate standard combination chemotherapy, platinum doublets (i.e., platinum plus paclitaxel, carboplatin plus gemcitabine, carboplatin plus liposomal doxorubicin), anti-vascular endothelial growth factor receptor antibodies (i.e., bevacizumab) combination therapy, poly (ADP-ribose) polymerase inhibitor (PARPi) (i.e., niraparib, olaparib) maintenance therapy, and secondary cytoreductive surgery (CRS) combined with chemotherapy. Nevertheless, the varied therapeutic effects of special choices are not mentioned in the guidelines for individuals, and the optimum treatment option is often accompanied by subjectivity which can differ in the different centers and relies on personal experience (Luvero et al., 2014).

Currently, based on a comprehensive evaluation of the efficacy and safety of drugs, it is feasible to select one treatment to increase therapeutic effect and reduce the body and financial burden on patients. Though several previous studies have made a direct comparison between a candidate drug and a placebo or another drug to obtain comparative efficacy and safety of the specific strategy for ROC, there is little published research comparing the efficacy of these various therapeutic regimens in patients with PSROC (Marchetti et al., 2016; Berg et al., 2019; Tomao et al., 2019). What is more, the excessive use of platinum may also cause some side effects for patients, like hypersensitivity reaction and residual toxicity of platinum, which are not discussed in the latest one. Are there some candidate options for this?

However, just using traditional pairwise meta-analysis methods to analyze this issue brings a challenge, the direct comparisons of certain treatments cannot be done due to a lack of evidence from head-to-head trials. Bayesian network meta-analysis is a potential solution to this problem. Bayesian network meta-analysis, an extension of traditional pairwise meta-analysis, allows performing the indirect comparison by linking a common comparator when a head-to-head trial is not available and enhances the inference on the relative efficacy of each treatment by including both direct and indirect evidence (Song et al., 2003; Ades et al., 2006; Sutton et al., 2008). Therefore, in this study, we aimed to do a Bayesian network meta-analysis of RCTs by integrating all available direct and indirect evidence to identify the best clinical choice for each patient.

## 2 Materials and methods

Meta-analysis was implemented according to the Preferred Reporting Items for Systematic Reviews and Meta-Analyses (PRISMA) statement (Hutton et al., 2015).

### 2.1 Information sources and search strategy

**FIGURE 1:**
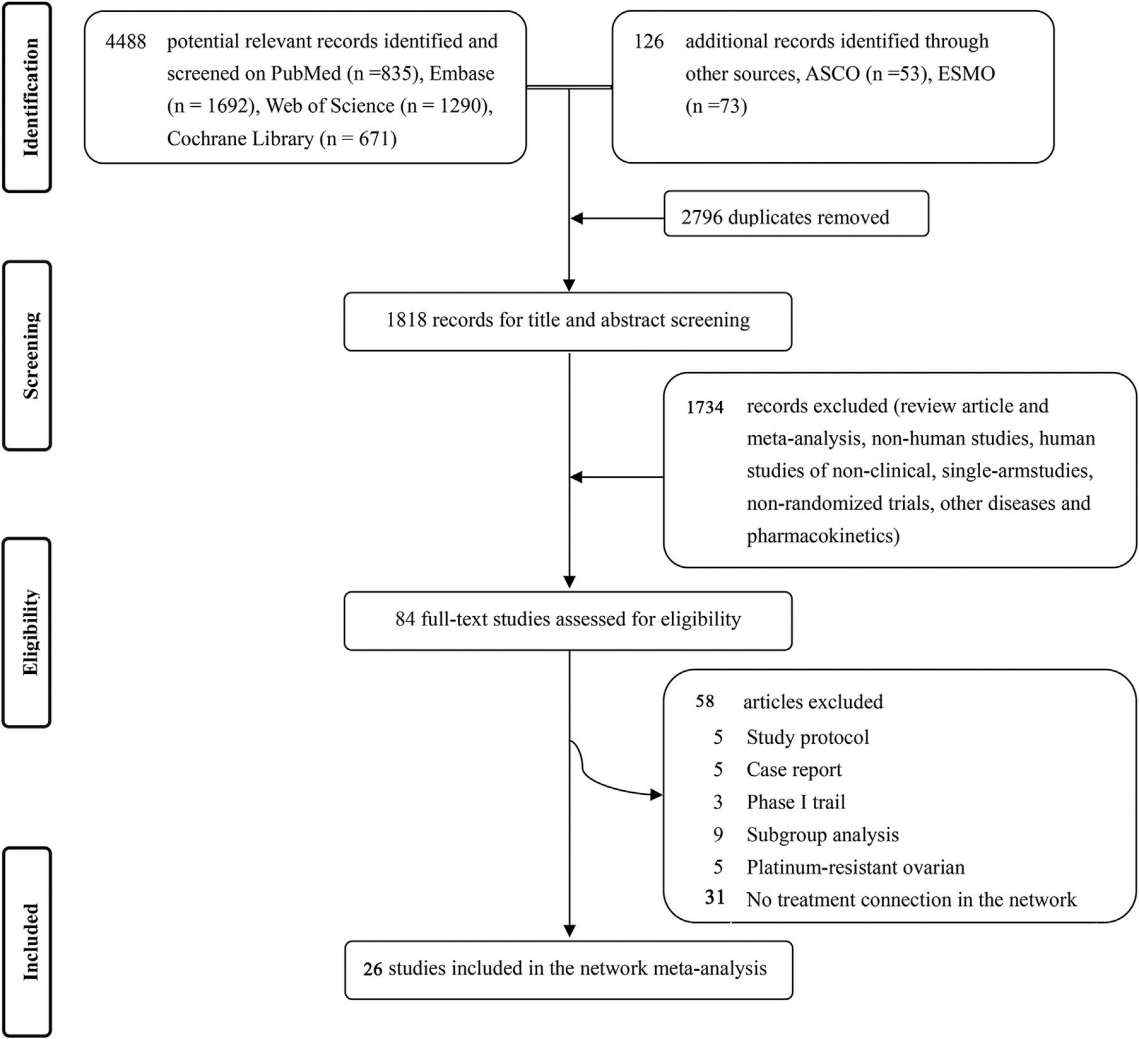
Flowchart of studies selection and design (PRISMA chart). ASCO = American Society of Clinical Oncology. ESMO = European Society of Medical Oncology.

Studies up to March 2022 were systematically searched on PubMed, Embase, Web of Science, and Cochrane Central Registry of Controlled databases with no language restrictions. A combination of the main searching items like “PSROC”, “chemotherapy” and “platinum” was used to find relevant randomized controlled trials (RCTs) and adjusted to adhere to the relevant rules in each database. Figure 1showed a PRISMA flow diagram. The detailed searching strategy is presented in Supplementary Appendix Table S1.

### 2.2 Study selection

Two independent reviewers (YL and YH) screened out titles and abstracts of all retrieved citations and examined potentially eligible studies in full text. RCTs were included if they met the following criteria:

1) Eligible patients were ≥18 years old with a histologically confirmed diagnosis of ovarian cancer and had disease progression ≥6 months following the last platinum-based chemotherapy regimen;
2) Patients with the measurable disease according to Response Evaluation Criteria in Solid Tumors or CA-125 assessable disease according to Gynecologic Cancer Inter Group criteria or histologically proven diagnosis of relapse;
3) Patients with an Eastern Cooperative Oncology Group performance status of ≤2; life expectancy of ≥12 weeks; and adequate bone marrow (granulocytes ≥2000/mm^3^, platelets ≥100 000/mm^3^), renal (creatinine clearance ≥40 ml/min), and hepatic (serum bilirubin and transaminases <1.5 upper normal limits) function;
4) Patients who had received maintenance biological therapy (e.g., bevacizumab) or hormonal therapy were eligible if it was completed at least 4 weeks after their last treatment;
5) Treatment of secondary cytoreduction: patients with the international model (iMODEL) score ≤ 4.7 or iMODEL score >4.7 accompanied by the serum level of cancer antigen 125 ≥ 10^5^ U/mL;
6) Trials reported measures on at least one of the following clinical outcomes: progression-free survival (PFS), overall survival (OS), objective response rate (ORR), adverse events (AEs) with grade 3 or higher, or specific AEs.

#### 2.2.1 Exclusion criteria

1) Patients with pre-existing neuropathy (National Cancer Institute Common Toxicity Criteria for Adverse Events grade >1);
2) If they had ovarian tumors of low malignant potential (borderline tumors);
3) Patients had received prior radiotherapy, or had a previous diagnosis of malignancy within the past 5 years (unless low risk of recurrence);
4) Vascular endothelial growth factor (VEGF) pathway–targeted therapy was restricted in bleeding diathesis or significant coagulopathy;
5) Other conditions like bowel obstruction, presence of symptomatic brain metastases, cardiopathy, severe active infection, and history of severe hypersensitivity reactions to compounds chemically related to study products.

### 2.3 Data extraction

Data parameters were extracted from the identified RCTs by CS and ST. The extracted information included: the first author, publication year, study ID, region, patient population under study, number of participants in each arm, patient age (median), characteristics of pharmaceutical intervention (dosage and duration of therapy) in each arm, and efficacy outcomes (PFS, OS, ORR, AEs, and treatment discontinuation for AEs). Adverse events related to treatment were selected with priority among all adverse events. The unpublished data were obtained from ClinicalTrials.gov and other available sources. Trial authors were also contacted when the important data were unclear or not displayed.

### 2.4 Risk of bias assessment

The risk of bias in eligible studies was assessed by the Cochrane risk-of-bias tool with predefined key domains of randomization, allocation concealment, blinding, data integrity assessment, and other sources of bias (Higgins et al., 2011). All studies were assessed under the same criteria.

Two reviewers (YL and JW) independently assessed the risk of bias in individual studies. Disagreement in the process of evaluation was resolved through consensus and arbitration by a panel of reviewers (YL, YH, SW, SA, and JW).

### 2.5 Data synthesis and statistical analysis

All direct and indirect evidence were synthesized to compare the efficacy and safety of therapeutic interventions, estimated hazard ratios (HR) with associated 95% credible intervals (CIs) for PFS and OS, odds ratios (OR) with associated 95% CIs for dichotomous outcomes (ORR, AEs, and treatment discontinuation for AEs). The primary outcome was PFS (the time from randomization to either death or disease progression, whichever came first), others were regarded as secondary outcomes including OS (the time between randomization and death from any cause), ORR according to RECIST 1.1 guidelines (the percentage of patients with a complete response or partial response), and AEs (specific treatment-related AEs or grade ≥ 3 AEs) through Common Terminology Criteria for Adverse Events (version 4.0) (Rustin et al., 2004). When duplicate publications were found, only trials with the most complete data of randomized control were included.

The Bayesian network meta-analyses were performed in WinBUGS software (version 1.4.3; MRC Biostatistics Unit, Cambridge, United Kingdom), based on Markov Chain Monte Carlo simulations (Lu and Ades, 2004). For further verification, results were reproduced by implementing R software (version 4.0.3) with package gemtc (version 0.8–8) and JAGS software (version 4.3.0). All outcomes were measured by a fixed-effects consistency model, as most direct evidence was from one trial (Zhao et al., 2019; Liu et al., 2021). Non-informative uniform and normal prior distributions were used, and three independent Markov chains were set to fit the model. For PFS and OS analysis, 150,000 sample iterations per chain were formed after 100,000 burn-ins and one step-size interval. For ORR, AEs and discontinue rate, parameters were modified by increasing sample iterations to 250,000, burn-ins to 150,000, and thinning interval to 10 to minimize autocorrelation. Convergence of iterations was evaluated using visualized trace plots and the Brooks-Gelman-Rubin, and finally, the posterior distributions of the model parameters (outcomes of the network meta-analysis) were output by this process (Brooks et al., 1998). The Bayesian approach also gave a ranking of treatments by calculating the surface under the cumulative ranking curves, listing each interventional strategy from the best to the worst according to its efficacy or safety (Cipriani et al., 2018).

Key assumptions underlying the network meta-analysis included transitivity (the similar and exchangeable for indirect comparisons) and consistency (the agreement between direct and indirect estimates) (Liu et al., 2021). Accordingly, a pairwise meta-analysis of included trials was performed by head-to-head comparisons, and the data was compared with that of the Bayesian framework for the evaluation of local inconsistency (Spiegelhalter et al., 2002). The inconsistency of the model was also estimated by the node-splitting approach, where direct and indirect evidence were separately compared on a special comparison (node). Heterogeneity between studies was measured by the I^2^ statistic, and the Cochrane Q test in a visual forest plot, the value of heterogeneity was considered mild, moderate, and severe heterogeneity (under 25%, between 25% and 50%, and over 50%, respectively) (Higgins et al., 2003). The subgroup analyses were performed according to PFI, BRCA mutation, and CRS. In addition, the transitivity of the included trials was calculated with a meta-regression analysis in STATA software (version 15.1). We generated network graphs in Stata (version 15) to elucidate treatments belonging to direct or indirect comparisons.

## 3 Results

### 3.1 Study selection

We identified 4,488 records from four databases and 126 additional online records from American Society of Clinical Oncology and European Society of Medical Oncology. After excluding the duplicates and non-pertinent studies, 84 studies were left for full-text review, and finally 26 trials (Parmar et al., 2003; González-Martín et al., 2005; Pfisterer et al., 2006; Alberts et al., 2008; Bafaloukos et al., 2010; Monk et al., 2010; Pujade-Lauraine et al., 2010; Aghajanian et al., 2012; Ledermann et al., 2012; Cognetti et al., 2013; Oza et al., 2015; Ledermann et al., 2016; Mirza et al., 2016; Coleman et al., 2017a; Coleman et al., 2017b; Pujade-Lauraine et al., 2017; Fujiwara et al., 2019; Mirza et al., 2019; Colombo et al., 2020; Pfisterer et al., 2020; Harter et al., 2021; Li et al., 2021; Pignata et al., 2021; Shi et al., 2021; Wang et al., 2021; Wu et al., 2021), met our eligibility criteria (Figure 1).

### 3.2 Study characteristics

The basic characteristics of the included studies were shown in Supplementary Appendix Table S2. A total of 6161 patients were enrolled in 10 different initial treatments: single-agent platinum (carboplatin or cisplatin), pegylated liposomal doxorubicin (PLD), carboplatin plus paclitaxel, carboplatin plus paclitaxel plus zibotentan, carboplatin plus paclitaxel plus bevacizumab, carboplatin plus gemcitabine, carboplatin plus gemcitabine plus bevacizumab, carboplatin plus PLD, carboplatin plus PLD plus bevacizumab; trabectedin plus PLD. A total of 4,280 patients were enrolled to receive four maintenance therapies: platinum-based chemotherapy (PBC), PBC plus bevacizumab, PBC plus cediranib, and PBC plus PARPi.

### 3.3 Risk of bias of included studies

**FIGURE 2:**
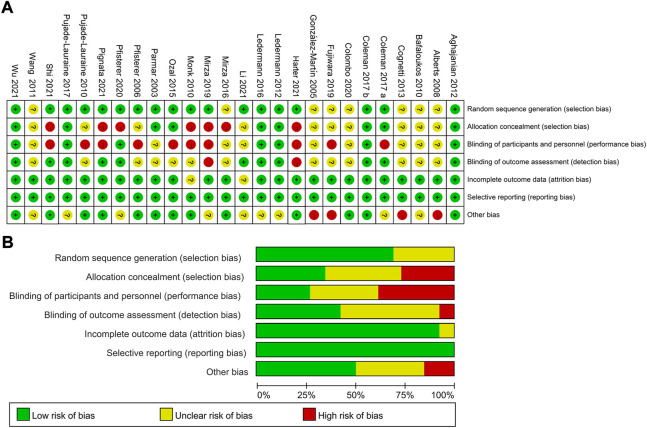
Risk assessment of bias using the Cochrane risk of bias. **(A)** Risk of bias items of all included studies are indicated as the percentages. **(B)** Each risk of bias item for each individual study. Green = low risk of bias, yellow = unclear risk of bias, red = high risk of bias.

**FIGURE 3:**
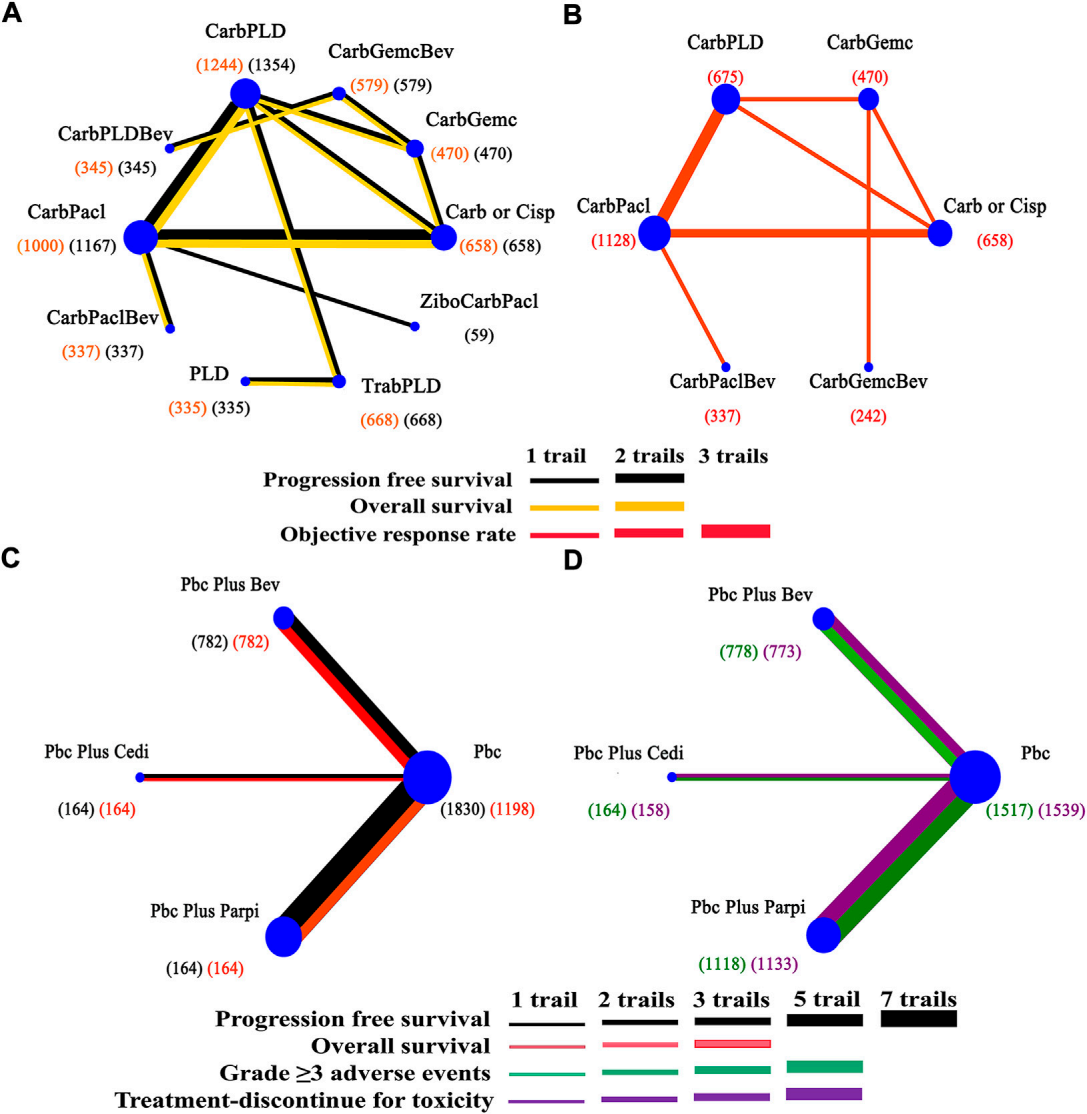
Network of interventional treatments in the Bayesian network meta-analysis. **(A)** Comparisons on progression free survival and overall survival in patients with platinum-sensitive ovarian cancer. **(B)** Comparisons on objective response rate. **(C)** Comparisons on progression-free survival and overall survival in maintain therapy. **(D)** Comparisons on adverse events of grade 3 or higher in maintain therapy. The size of each circle is proportional to the total number of patients receiving a treatment. The width of lines is proportional to the number of studies comparing the connected treatments.

Figure 2 exhibited the detailed risk of bias assessments according to the Cochrane’s Collaboration risk of bias tool, suggesting nearly half of the trials were open-label and therefore potentially affected by performance bias. The Egger regression test was performed to determine the publication bias and a *p*-value >0.05 suggested no publication bias in the included studies (Supplementary Appendix Figure S1). The network plots were presented in Figure 3. For initial treatments, bevacizumab combination therapy, except carboplatin-PLD-bevacizumab, was made a direct comparison with the respective platinum doublet therapy, and all platinum doublet therapies were directly compared with single-agent platinum. For maintenance treatments, only randomized, controlled clinical trials were included in this study to minimize the heterogeneity.

### 3.4 Network meta-analysis by initial treatments of PSROC

Network meta-analysis included all treatments for PFS, 12 treatments for OS (Figure 3A), nine treatments for ORR, and 13 treatments for AEs (Figure 3B). Data was extracted from trials (Parmar et al., 2003; González-Martín et al., 2005; Pfisterer et al., 2006; Alberts et al., 2008; Bafaloukos et al., 2010; Monk et al., 2010; Pujade-Lauraine et al., 2010; Aghajanian et al., 2012; Ledermann et al., 2012; Cognetti et al., 2013; Oza et al., 2015; Ledermann et al., 2016; Mirza et al., 2016; Coleman et al., 2017a; Coleman et al., 2017b; Pujade-Lauraine et al., 2017; Fujiwara et al., 2019; Colombo et al., 2020; Pfisterer et al., 2020; Li et al., 2021; Pignata et al., 2021; Wang et al., 2021; Wu et al., 2021).

**FIGURE 4:**
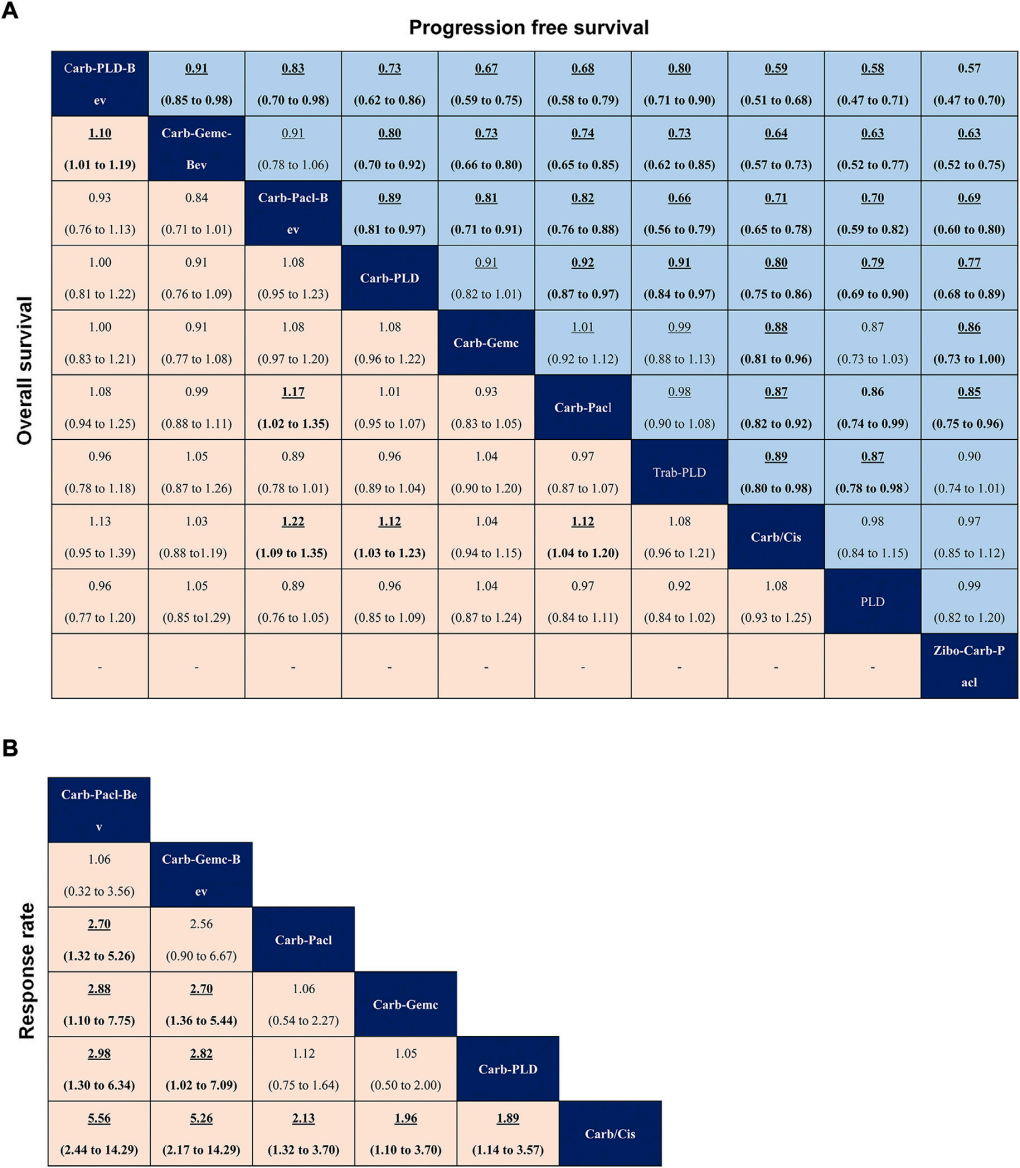
Pooled estimates of initial treatments in the network meta-analysis. **(A)** Data are hazard ratios for progression-free survival (upper triangle) and overall survival (lower triangle). **(B)** Odds ratios for objective response rate. Data with hazard or odds ratios represent the comparison of row-defining treatment versus column-defining treatment. Data in parentheses are the 95% credible intervals. Hazard ratios less than one and odds ratios more than one favour the row-defining treatment. Significant results are highlighted in bold and underline.

In terms of PFS (Figure 4A), patients who received the bevacizumab combination were more likely to obtain greater PFS benefits than those who received dual combination therapies or single-agent platinum, and carboplatin-PLD-bevacizumab provided the best PFS benefit [carboplatin-gemcitabine-bevacizumab *vs.* (HR 0.91, 95% credible interval 0.85–0.98), carboplatin-paclitaxel-bevacizumab *vs.* (0.83, 0.70–0.98)]. Dual combination chemotherapy was found to yield superior PFS benefits than single-agent platinum. PLD provided similar PFS to single-agent platinum (0.98, 0.84–1.15), but zibotentan-carboplatin-paclitaxel seemed to show a deficiency in prolonging PFS, compared to carboplatin/cisplatin (0.97, 0.85–1.12).

In terms of OS (Figure 4A), carboplatin-paclitaxel-bevacizumab had a better advantage in improving OS when compared with other treatments (carboplatin-gemcitabine, carboplatin-paclitaxel, carboplatin/cisplatin). Except for carboplatin-gemcitabine, dual combination therapies (carboplatin-PLD, carboplatin-paclitaxel) produced better OS than single-agent platinum. Carboplatin-PLD-bevacizumab significantly prolonged OS compared with carboplatin-gemcitabine-bevacizumab (HR 1.10, 95% credible interval 1.01–1.19), although both of which were shown to have similar efficacy versus single-agent platinum.

In terms of ORR (Figure 4B), the efficacy corresponded roughly to the results of PFS and OS, whereas the bevacizumab combination consistently revealed better ORR than other classes. Among them, carboplatin-paclitaxel-bevacizumab was the best one and single-agent platinum was the worst in the network analysis (carboplatin-paclitaxel-bevacizumab versus carboplatin/cisplatin: OR 5.56, 95% credible interval 2.44–14.29). Interestingly, carboplatin-gemcitabine-bevacizumab and carboplatin-paclitaxel seemed to have similar ORR (2.56, 0.90–6.67), but there were no significant differences between carboplatin-paclitaxel and other dual combination therapies.

In terms of safety, since the overall toxicity was not reported in most studies, 10 specific AEs were chosen from the most clinically relevant events in the current study. Commonly reported AEs included nausea and vomiting, fatigue, alopecia, allergy, diarrhea, neuropathy, neutropenia grade ≥3, thrombocytopenia grade ≥3, and anemia grade ≥3 (Supplementary Appendix Figure S2). Carboplatin-PLD-bevacizumab showed the greatest probability to cause fatigue, nausea, vomiting, and diarrhea, followed by other bevacizumab combinations. Carboplatin-gemcitabine-bevacizumab was associated with the highest risk of thrombocytopenia grade ≥ 3, and carboplatin-PLD-bevacizumab resulted in more frequent alopecia and allergy. Dual combination therapies had relatively mild toxicity spectrums, and stomatitis was predominant in carboplatin-PLD, more neutropenia grade ≥ 3 in carboplatin-gemcitabine, and more neuropathy in carboplatin-paclitaxel. Zibotentan-carboplatin-PLD caused more alopecia and single-agent platinum was the narrowest and safest drug treatment.

### 3.5 Network meta-analysis by maintenance therapy of PSROC

A total of 11 RCTs were included in the maintenance therapy. Outcomes for estimation were PFS, OS, AEs of grade 3 or higher, and treatment discontinuation for toxicity in network meta-analyses. Four therapeutic strategies were available for all comparisons (Figure 3C). Data was extracted from trials (Aghajanian et al., 2012; Ledermann et al., 2012; Oza et al., 2015; Ledermann et al., 2016; Mirza et al., 2016; Coleman et al., 2017a; Coleman et al., 2017b; Pujade-Lauraine et al., 2017; Li et al., 2021; Pignata et al., 2021; Wu et al., 2021).

**FIGURE 5:**
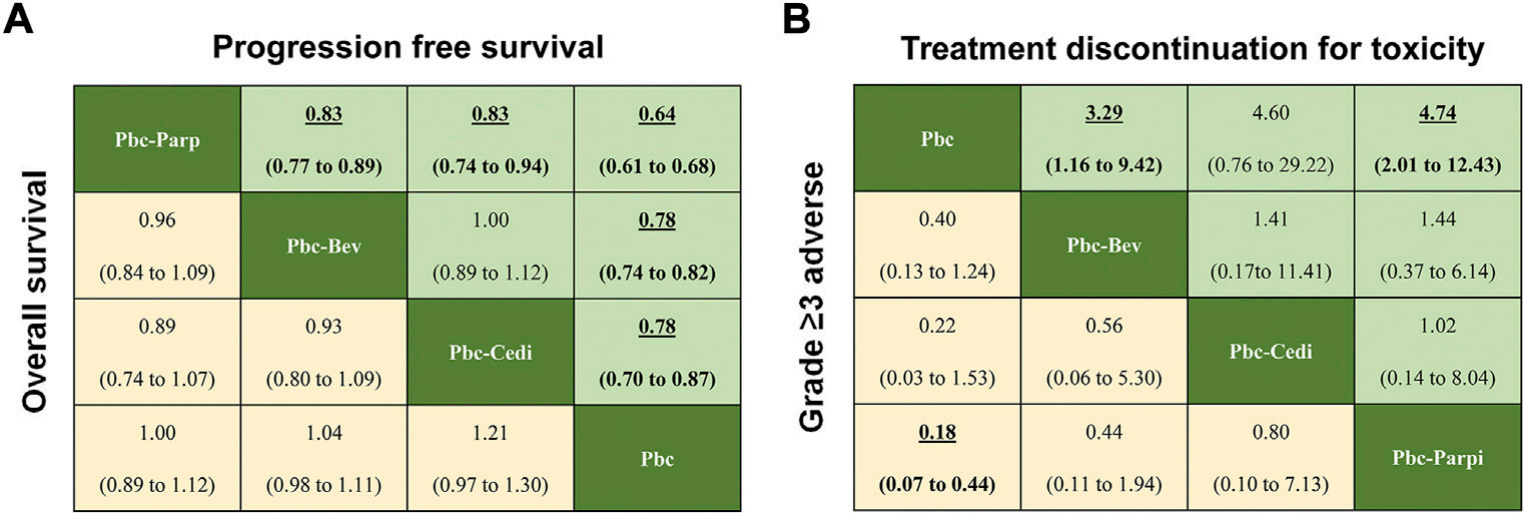
Pooled estimates of maintain therapies in the network meta-analysis. **(A)** Pooled hazard ratios for progression-free survival (upper triangle) and overall survival (lower triangle). **(B)** Odds ratios for treatment discontinuation for toxicity (upper triangle) and adverse events of grade three or higher (lower triangle). Data in parentheses are the 95% credible intervals. Hazard ratios less than one and odds ratios more than one favour the row-defining treatment. Significant results are Highlighted in bold and underline.

Regarding PFS of maintenance treatments (Figure 5A), the significant differences in PBC-PARPi versus PBC-bevacizumab (HR 0.83, 95% credible interval 0.77–0.89), PBC-cediranib (0.83, 0.74–0.94), and PBC (0.64, 0.61–0.68), were in favor of PBC-PARPi as the best response to PSROC. Of note, other maintenance treatments also significantly increased PFS when compared with PBC, and the difference was marginal in PBC-bevacizumab versus PBC-cediranib (1.00, 0.89–1.12). In terms of the OS (Figure 5A), no significant difference was observed in any two comparisons, as most hazard ratios were close to 1.

Regarding safety (Figure 5B), the addition of maintenance drugs to platinum-based chemotherapy was associated with a rise in the risk of AEs of grade 3 or higher. We saw a possibly increased toxicity in patients with PBC-bevacizumab (OR 0.40, 95% credible interval 0.13–1.24), PBC-cediranib (0.22, 0.03–1.53) than PBC, but PBC-PARPi had a significantly higher rate of AEs of grade 3 or higher in comparison with PBC (0.18, 0.07–0.44). A similar situation occurred in toxicity-related treatment discontinuation a higher discontinue rate was observed in PBC-bevacizumab (3.29, 1.16–9.42) and PBC-PARPi (4.74, 2.01–12.43) than in PBC, but there was no significant difference among maintenance therapies.

### 3.6 Subgroup analysis of PFS

**FIGURE 6:**
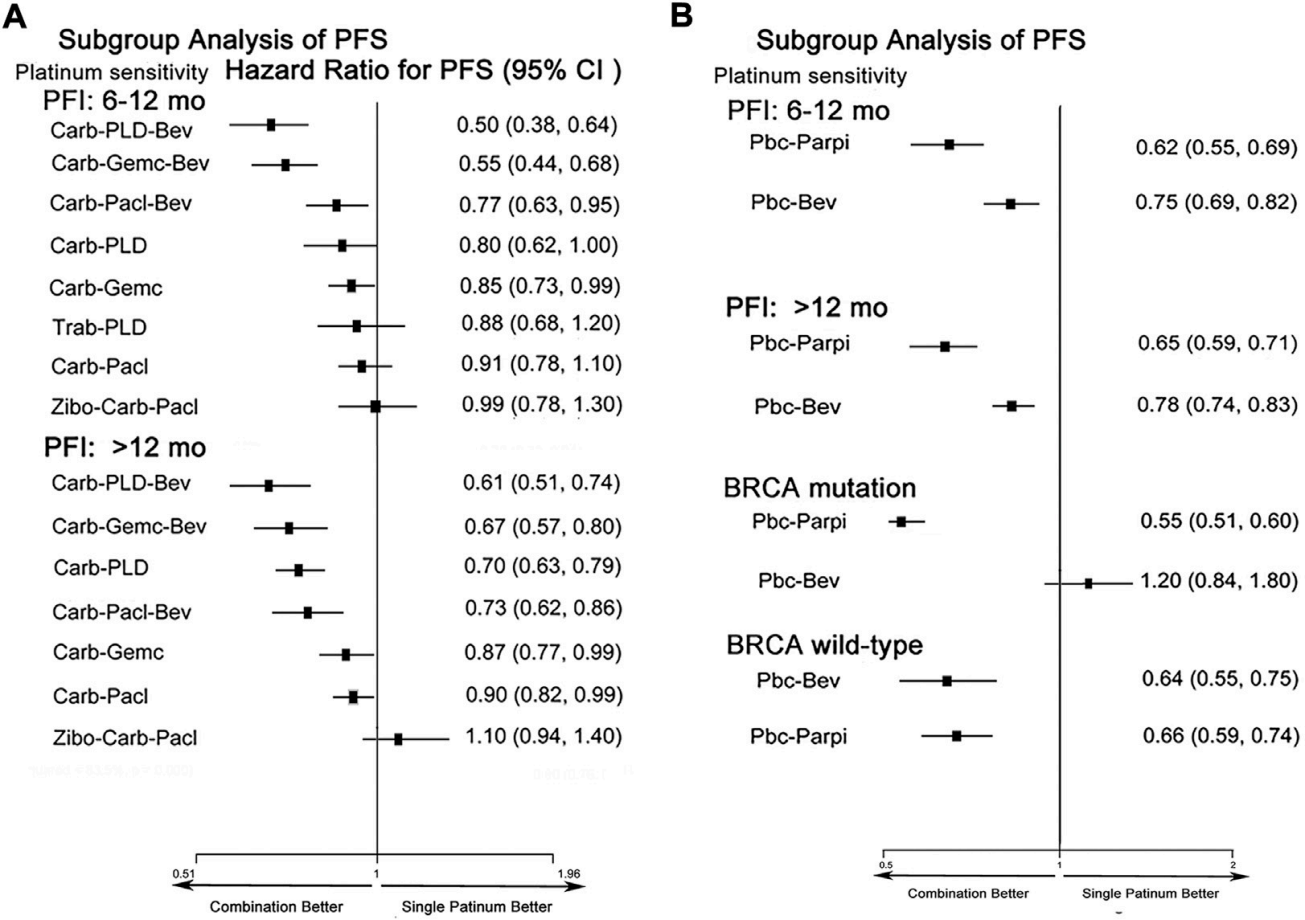
Forest plots of subgroup analysis of progression-free survival in patients with PSROC. **(A)** Hazard ratios and 95% CIs for progression-free survival in initial treatments. **(B)** Hazard ratios and 95% CIs for progression-free survival in maintenance treatments. Hazard ratios <1.00 provide a better survival benefit.

We evaluated PFS by PFI and BRCA mutation (Figure 6). In initial treatments, bevacizumab combined with platinum doublets showed superiority in PSROC regardless of PFI. Trabectedin-PLD also provided an equal PFS with single platinum in the PFI (6–12 months). Remarkably, for patients with the PFI (>12 months), compared to carboplatin/cisplatin, their hazard risk of PFS in the platinum doublets developed a left shift to favor survival. Regarding maintenance therapy, both PBC-PARPi and PBC-bevacizumab significantly extended PFS more than PBC. Interestingly, BRCA mutation could support the benefit of PFS of patients with PBC-PARPi but lead to a poor PFS for PBC-bevacizumab and lower than PBC. To confirm the negative PFS of bevacizumab in BRCA-mutated PSROC patients, we compared the PFS of bevacizumab combination with that of chemotherapy alone (Aghajanian et al., 2012; Coleman et al., 2017a; Mirza et al., 2019; Pignata et al., 2021). A similar result was obtained (HR 0.88, 95% credible interval 0.28–2.77) (Supplementary Appendix Figure S3A). Also, secondary cytoreduction followed by chemotherapy had significantly longer PFS than chemotherapy alone in patients with PSROC (Supplementary Appendix Figure S3B).

### 3.7 Rank probabilities

**FIGURE 7:**
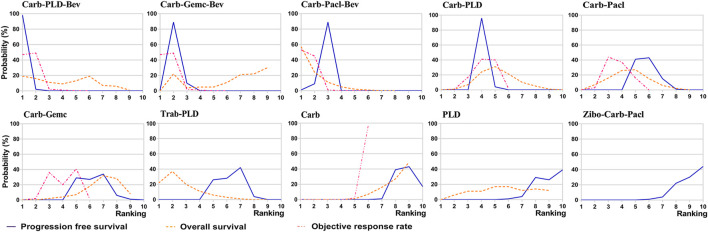
Bayesian ranking curves of initial treatments based on efficacy. The probability of each comparable treatment ranking from first to last on progression-free survival, overall survival, objective response rate.

**FIGURE 8:**
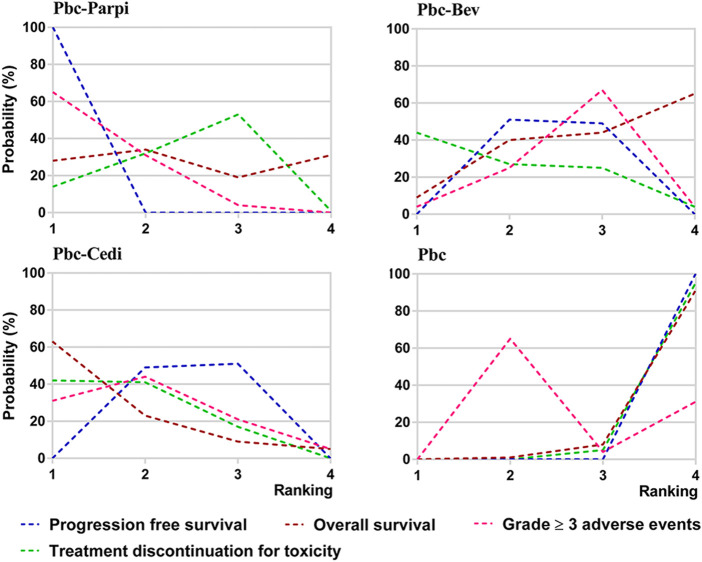
Bayesian ranking curves of maintain therapies on efficacy. The probability of each comparable treatment being ranked from first to last on progression-free survival, overall survival, grade ≥ 3 adverse events, and treatment discontinuation for toxicity.

Ranking analysis of comparable treatments was performed through the Bayesian ranking profiles. The ranking results were almost in accord with the pooled analyses of hazard and odds ratios. For the initial treatments of PSROC patients (Figure 7), carboplatin-PLD-bevacizumab most likely ranked first in PFS analysis (cumulative probability of 98%), and carboplatin-paclitaxel-bevacizumab led in both OS (57%) and ORR (53%). Single-agent platinum and zibotentan-carboplatin-paclitaxel had the highest probabilities of ranking last in the estimated results, with the worst PFS (44% in zibotentan-carboplatin-paclitaxel), OS (49% in single-agent platinum), and ORR (97% in single-agent platinum). When the maintenance setting for PSROC was considered (Figure 8), PBC-PARPi had the greatest probability of being ranked first in PFS (100%) and AEs of grade 3 or higher (65%), whereas PBC-cediranib accounted for 63% in OS, PBC-bevacizumab and PBC-cediranib accounted for 44% and 42% in AEs related discontinuation, respectively. On the contrary, PBC had lower efficacy but better safety, ranking last in PFS (100%), OS (67%), but safest in AEs of grade 3 or higher (91%), and AEs-related discontinuation (95%).

### 3.8 Heterogeneity and inconsistency assessment

The heterogeneity of pairwise comparisons was evaluated. Outcomes showed that most comparisons had low or moderate heterogeneity (I^2^<50%) in the included studies (Supplementary Appendix Figure S4). However, high heterogeneity was also observed in comparisons like carboplatin-PLD vs. carboplatin-paclitaxel in PFS (I^2^ = 67%), carboplatin-paclitaxel vs. carboplatin in OS (I^2^ = 82%), and PBC-bevacizumab *vs.* PBC in PFS (I^2^ = 52%). Inconsistency between direct and indirect comparisons was estimated by the node-splitting analysis and the data did not show significant differences in the reported outcomes (PFS, OS, and ORR) (Supplementary Appendix Table S3, and the evidence from the pairwise meta-analysis was almost in line with that from network meta-analysis.

## 4 Discussion

### 4.1 Principal findings

In the present systematic review and network meta-analysis, we comprehensively assessed the relative efficacy and safety of currently available treatments, mainly focused on platinum-based chemotherapy for patients with PSROC. Our work could provide support for better clinical choices, and the results included the following:

1) Bevacizumab combined with platinum doublet had a better survival superiority over standard chemotherapy regarding OS, PFS, and ORR except for BRCA mutated patients; PARPi showed a potential advantage over antiangiogenic agents in the maintenance treatment stage.
2) As an individual treatment, carboplatin-PLD-bevacizumab provided the best PFS, while carboplatin-paclitaxel-bevacizumab consistently resulted in the best OS and ORR for patients with PSROC, they were associated with different toxicity spectrums.
3) The addition of PARPi to PBC was the most promising treatment in prolonging PFS, but all maintenance strategies (PBC-bevacizumab, PBC-cediranib, and PBC-PARPi) failed to effectively improve OS compared with PBC.
4) Maintenance therapy caused more toxicity in general when compared with standard chemotherapy, especially when PARPi was selected as the subsequent medical treatment after PBC.
5) CRS plus chemotherapy was more prone to offer a PFS benefit for PSROC patients than chemotherapy.

### 4.2 Comparison with existing literature

Accumulated evidence showed that single-agent platinum drug was widely used in platinum-sensitive relapsed ovarian cancer due to excellent acceptability, convenience, and high response rates, especially when the treatment-free interval was over 24 months and the patient’s response rate was nearly 60% after re-treatment with platinum (Ozols, 2005). However, to improve survival outcomes, combination therapy had become increasingly important in treating PSROC. We observed that multiple chemotherapies provided significantly better survival benefits than carboplatin or cisplatin alone, which contributed to the fact that ovarian cancer progress could be further inhibited by the combination of agents with different mechanisms of action.

Bevacizumab, as an adjunctive therapy with platinum doublets in PSROC, enhanced the efficacy in suppressing the growth of tumors. One of the symbols of ovarian cancer was massive angiogenesis formation that promoted tumor proliferation and metastasis, but bevacizumab could induce the antiangiogenic effect by directly targeting VEGF (Shoji et al., 2019). By depleting regulatory T cells, bevacizumab also exerted multipronged immunostimulatory functions for various tumors (Napoletano et al., 2019). Hence, the efficacy of platinum-based chemotherapy might be boosted through the addition of bevacizumab to reverse VEGF-mediated neoangiogenesis and immunosuppression.

In this study, carboplatin-paclitaxel-bevacizumab appeared to have the best OS and ORR in the initial treatment, which might be because of the multipoint anticancer effect of the combination chemotherapy, and bevacizumab also increased the paclitaxel concentration in tumor owing to the downregulation of vascular permeability with no change in paclitaxel concentration of the plasma or liver (Yanagisawa et al., 2010). We also found carboplatin-PLD-bevacizumab presented excellent superiority in PFS since PLD with a special size (approximately 100 nm) could infiltrate through neovascularization vessels into the tumor without any impact on normal vessels, and bevacizumab might encourage this process (Green and Rose, 2006). Moreover, the MITO16B trial indicated that patients with PSROC (PFI >6 months) could get further improvement in PFS of 3 months by treatment with bevacizumab combination with platinum-based chemotherapy, even if they had received bevacizumab previously (Pignata et al., 2018).

Meanwhile, the effect of bevacizumab seemed to be weakened in patients with BRCA gene mutation. Our results showed that compared to chemotherapy alone, bevacizumab combined with chemotherapy failed to improve the PFS of BRCA-mutated PSROC patients, and this was consistent with findings from a large case-control study of advanced ovarian cancer (Lorusso et al., 2020). PAOLA1 trial recommended the combination of olaparib and Bev for BRCA mutation ovarian cancer according to the increased PFS superiority than to the Bev monotherapy arm (Ray-Coquard et al., 2019). However, this study lacked a comparison between olaparib plus Bev and olaparib alone, so that the benefit of Bev could not be definitively confirmed for BRCA mutation patients. Recently, according to the joint analysis of SOLO1 and PAOLA-1 trials, population-adjusted indirect treatment comparison between olaparib plus Bev and olaparib alone treated BRCA-mutated patients showed a 24-month PFS of 82% and 72%, respectively (HR 0.71, CI 95% 0.45–1.09), and Bev did not seem to provide a significant survival benefit for BRCA-mutated patients (Vergote et al., 2021).

An explanation of these findings could be associated with the tumor microenvironment (Farolfi et al., 2020). In fact, BRCA1 had a function in regulating VEGF synthesis as a response to hypoxic conditions, and the accumulation of HIF-1α also declined after BRCA gene KO even in the hypoxia environment (Kang et al., 2006). In addition, the tumor with BRCA disruption had a high frequency of developing into immuno-reactive subtypes, especially the tumor-infiltrating lymphocytes. By contrast, a stromal or mesenchymal ovarian cancer focused on the activation of angiogenesis-related genes rather than immune-related genes (Kommoss et al., 2017). Therefore, it could be speculated that bevacizumab had low activity in BRCA-mutated ovarian cancer, BRCA test results should be obtained for the bevacizumab application. Well-designed large randomized controlled trials were also required to support this point.

Interestingly, we observed the non-platinum combination (trabectedin-PLD) had similar PFS to single platinum for patients when the PFI was from 6 to 12 months. It was reported that trabectedin has dual effects on anticancer including inducing the differentiation and apoptosis of malignant cells and regulating the tumor microenvironment by limiting the associated inflammatory mediator production such as CCL2, interleukin-6, and VEGF (Poveda et al., 2014; Ventriglia et al., 2018). Corresponding with our finding, the randomized phase III OVA-301 trial also suggested that trabectedin-PLD could give an OS benefit. Patients diagnosed with PSROC had an 18% decrease in the risk of death in trabectedin-PLD compared with PLD, and the OS benefit was enhanced for patients with the partially platinum-sensitive disease (PFI of 6–12 months) (Poveda et al., 2011). Importantly, the hazards of residual toxicity or hypersensitivity reactions caused by platinum or taxane agents restricted their reuse for PSROC, and trabectedin plus PLD might contribute to delaying the PFI extension for patients with a partially respond rate to recover from the toxicity of the last platinum-based therapy, which enabled the subsequent platinum.

Although platinum-based regimens gave good remission rates for patients with PSROC, the majority would suffer from recurrent disease progression in the end. Drugs for maintenance therapy presented advantages to extend the PFS interval. In this study, we found that PBC-PARPi provided the best PFS benefit for PSROC patients with or without BRCA mutation. Possible explanations were that PARPi inhibitors interrupted the repair of DNA single-strand break mediated by poly (ADP-ribose) polymerase enzyme and this process induced tumor synthetic lethality (Farmer et al., 2005; Berek et al., 2021). Interestingly, homologous recombination could sustain paired double-stranded DNA in the presence of PARPi, but homologous recombination deficiency was commonly observed in ovarian cancer, especially for the high-grade serous (Lord and Ashworth, 2016). Thus, the benefit of PARPi might become more obvious for PSROC. Another reason was that PARPi could lock the poly (ADP-ribose) polymerase enzyme on the DNA to prevent DNA replication of cancer cells (Shen et al., 2015). PARPi monotherapy (rucaparib) was also approved to treat patients with a deleterious BRCA mutation when they failed to respond to at least two prior lines of platinum-based chemotherapy (Bouberhan et al., 2019; Lee and Matulonis, 2020). However, AEs of grade 3 or higher were more common in PBC-PARPi in the network analysis but did not lead to treatment discontinuation in most patients. In general, maintenance therapy following platinum-based chemotherapy had made improvements to PFS for patients with PSROC, which delayed the requirements to implement subsequent lines of cytotoxic chemotherapy.

However, according to our findings, all maintenance regimens (bevacizumab, cediranib, and PARPi) accompanied with PBC did not significantly improve OS when compared with PBC alone. It might be because of the requirement for longer follow-up time since the data from most studies were insufficient to evaluate OS, and the maturity of three-quarters of included trials was less than 60%, which might bring an incorrect assessment due to the mixture effects of receiving subsequent chemotherapy on overall survival. Therefore, PFS was still considered as the primary endpoint to evaluate the effectiveness of drugs for ROC.

### 4.3 Strengths and limitations

Our evidence could supplement recent guidelines about how to effectively use platinum-based combinations and maintenance therapy for PSROC individuals according to platinum-sensitive grade, and which treatments might be the most promising regimens to follow. Individual treatment is receiving enhanced attention, and rationally choosing agents ensures an efficient cure rate for patients when facing limited medicines.

There were several limitations in the present network meta-analysis. Firstly, most comparisons of treatments were based on indirect evidence and most head-to-head analysis was pooled from one trial, which might lead to a risk of imprecision. Thus, to increase the reliability of our outcomes, we extracted all data from randomized controlled studies, and also did an inconsistency, transitivity, and risk of bias assessment to confirm the reasonability of this study.

Secondly, accumulated information indicated that the BRCA status of patients was associated with the effect of platinum-based therapy on PSROC. In this study, the majority of included studies failed to report complete baseline characteristics due to a lack of knowledge of the correlation between the biomarkers and drugs. Patients with BRCA status were more likely to obtain survival benefits from platinum and PARPi treatments, but interestingly, BRCA1 or BRCA2 mutations of patients could be reversed after exposure to these two agents (Sakai et al., 2008; Swisher et al., 2008; Weigelt et al., 2017). Whether this reversion would lead to drug resistance in BRCA mutation carriers needed to be further studied, especially for PARPi, which had shown the best treatment when combined with platinum regimens in the network. Moreover, BRCA mutation seemed to disable the benefit of bevacizumab beyond progression (Figure 6, Supplementary Appendix Figure S3). Currently, whether the effect of other treatment options will be affected by BRCA status is unknown.

Thirdly, patients were not stratified according to CRS, which might cause heterogeneity in treatment benefits (Lee et al., 2015; Harter et al., 2021; Shi et al., 2021). Three RCTs had shown that CRS combined with chemotherapy provided a significantly longer PFS (approx. 7 months PFS increase) for patients with PSROC than chemotherapy alone, and the benefit of bevacizumab maintenance was enhanced by CRS. However, most trials in this study did not report the relevant detail, GOTIC003 study restricted surgical therapy for eligible patients, and the percentage of patients with CRS varied from 10% to 60% among AGO-OVAR2.21, GOG-0213, OCEANS, CALYPSO, and NORA study, although the number of the special patients was equally assigned to the experimental and control group.

## 5 Conclusion

In our analysis of women with PSROC, platinum combination treatments significantly improved survival rates and had comparable safety profiles. In terms of efficacy, PBC-PARPi and PBC-bevacizumab provided a better PFS over other treatments, and PBC-PARPi rather than PBC-bevacizumab was preferentially recommended as BCRA mutation occurring in clinic practice. In long term, patients might get benefits from single-platinum agents, platinum-based combinations, or the non-platinum regimen, and all of which relied on the platinum-sensitivity status of patients. These findings could complement the current standard of care and give a reference to design future trials, like PBC-PARPi versus PBC-bevacizumab, and carboplatin-PLD, carboplatin-gemcitabine or carboplatin-paclitaxel versus trabectedin-PLD for PSROC.

## Data Availability

The original contributions presented in the study are included in the article/Supplementary Material, further inquiries can be directed to the corresponding authors.
