# Supplementary material for: A comprehensive comparison of medication strategies for platinum-sensitive recurrent ovarian cancer: A Bayesian network meta-analysis
Source: Front Pharmacol. 2022 Nov 10;13:1010626. doi: 10.3389/fphar.2022.1010626 (PMC9691266; doi:10.3389/fphar.2022.1010626)
Supplement: Supplementary file 2 [file DataSheet1.PDF]

Supplementary Material

Supplement: Additional Tables and Figures

We used the following search terms: (recurrent [title/abstract] OR relapse [title/abstract]) AND (high-grade serous [title/abstract] OR endometrioid disease[title/abstract] OR fallopian tube [title/abstract] OR ovarian [title/abstract])) AND platinum [title/abstract] AND sensitive [title/abstract] AND (chemotherapy [title/abstract]) AND ("0001/01/01"[Date - Publication] : "2022/3/12"[Date - Publication]). No language limitations were adopted.

Appendix Table S1. Literature search strategy

First author, year	Study ID	Phase	Sample size (no); median age	Intervention arm	Control arm	Reported outcomes
Pfisterer et al 2020	AGO-OVA R2.21 NCT01837251	III, multiple	337/345<br>63/62	Bev (15 mg/kg, day 1) plus carb (AUC=4 plus Gemc (1,000 mg/m^2, day 1 and 8) every 3 wks (6 cycles) plus maintenance Bev (15 mg/kg)	Bev (10 mg/kg, day 1 and 15) (6 cycles) plus Carb (AUC=5, day 1) plus PLD (30 mg/m^2, day 1) every 4 wks plus maintenance Bev (15 mg/kg)	PFS, OS, AEs
Coleman et al 2017	GOG-0213 NCT00565851	III, multiple	337/337<br>59.5/60.6	Pacl (175 mg/m^2) plus Carb (AUC=5) plus Bev (15 mg/kg) every 3 wks plus maintenance Bev (15 mg/kg)	Pacl (175 mg/m^2) plus Carb (AUC=5) every 3 wks (6 cycles)	PFS, OS, ORR, AEs
Aghajanian et al 2012	OCEANS	III, multiple	242/242<br>60/61	Gemc (1,000 mg/m2, days 1 and 8) plus Carb (AUC=4, day 1) plus Bev (15 mg/kg, day 1) every 3 wks (6-10 cycles) plus maintenance Bev (15 mg/kg)	Gemc (1,000 mg/m^2, days 1 and 8) plus Carb (AUC=4, day 1) plus placebo (15 mg/kg, day 1) every 3 wks (6-10 cycles) plus maintenance Placebo (15 mg/kg)	PFS, OS, ORR, Selected AEs
Pujade-Lauraine et al 2010	CALYPSO	III, multiple	466/507<br>60.5/61	PLD (30 mg/m^2, day 1) and Carb (AUC=5, day 1) every 4 wks (≥6 cycles)	Pacl (175 mg/m^2, day 1) and Carb (AUC=5, day 1) every 3 wks (≥6 cycles)	PFS, OS, AEs
Bafaloukos et al 2010	ACTRN12609000436	II, Greece	93/96<br>62/63	Carb (AUC=5, day 1) plus PLD (45 mg/m^2, day 1)	Carb (AUC=5, day 1) plus paclitaxel (175 mg/m^2, day	Time-to-progression[a],

	279			every 28 days (6 cycles)	1) every 21 days (6 cycles)	OS[a], ORR, AEs
Wang et al 2011	NCT04337632	III, multiple	110/106<br>56/55	Carb (AUC=5, day 1) plus PLD (25 mg/m^2, day 1) every 28 days (6 cycles)	Carb (AUC=5, day 1) plus Pacl (175 mg/m^2, day 1) every 21 days (6 cycles)	PFS, ORR, AEs
Pfisterer et al 2006	AGO-OVAR	III, multiple	178/178<br>59/58	Gemc (1,000 mg/m^2, days 1 and 8) and Carbo (AUC=4, day 1) every 3 wks (6-10 cycles)	Carb (AUC=5, day 1) every 3 wks (6-10 cycles)	PFS, OS, ORR, selected Aes
Alberts et al 2008	SWOG-0200	III, USA	31/30<br>66.9/62.5	PLD (30 mg/m^2) plus Carb (AUC=5) every 4 wks (7 cycles)	Carb (AUC=5) every 4 wks (6 cycles)	PFS, OS, ORR, AEs
González-Martín et al 2005	GEICO-0199	II, Spanish	38/40<br>59/61	Pacl 175 mg/m^2 plus Carb (AUC=5) every 3 wks (6-9 cycles)	Carb (AUC=5) every 3 wks (6-9 cycles)	PFS, OS, ORR, AEs
Fujiwara et al 2019	GOTIC003	II, multiple	49/50<br>NA/NA	PLD (30 mg/m^2) plus Carb (AUC=5) every 4 wks (≥6 cycles)	Gemc (1,000 mg/m^2 on days 1 and 8) plus Carb (AUC=4) every 3 wks (≥6 cycles)	PFS, OS, ORR, AEs
Colombo et al 2020	INOVATYON NCT01379989	III, multiple	331/306<br>NA/NA	Trab (1.1 mg/m^2) plus PLD (30 mg/m^2) every 4 wks (6 cycles)	Carb (AU=5) plus PLD (30 mg/m^2) every 4 wks (6 cycles)	PFS, OS, Selected AEs
Parmar et al 2003	ICON4	III, multiple	392/410<br>60.0/59.2	Carb (AUC=5 or 6) or Cisp (50 mg/m^2) plus Pacl (175 mg/m^2 or 185 mg/m^2) every 3 wks (6-8 cycles)	Carb (AUC=5 or 6) or Cisp (75 mg/m^2) every 3 wks (6-8 cycles)	PFS, OS, ORR, AEs
Cognetti et al 2013]	AGO-OVAR 2.14 NCT00929162	II, multiple	59/61<br>57.4/56.6	zibotentan (10 mg once-daily) plus Pacl (175 mg/m^2) plus Carb (AUC=5) every 3 wks (≤8 cycles)	Placebo (once-daily) plus Pacl (175 mg/m^2) plus Carb (AUC=5) every 3 wks (≤8 cycles)	PFS, AEs
Monk et al 2010	NCT00113607	III, multiple	218/213<br>56.0/58.0	PLD (30 mg/m^2) followed by a 3-hour infusion of Trab (1.1 mg/m^2) every 3 wks	PLD (50 mg/m^2) every 4 wks	PFS, OS
Wu et al 2021	NORA NCT03705156	III, multiple	177/88<br>53/55	Pbc[b] plus bodyweight ≤77 kg and a platelet count ≤150 × 10^3/μL niraparib	Pbc[b] plus matched placebo in 28 days cycles until disease progression	PFS, OS, grade ≥3 AEs, TDT

				300 mg/day orally; otherwise, niraparib 200 mg/day orally in 28 days cycles until disease progression		
Mirza et al 2016	ENGOT-OV16 NCT01847274	III, multiple	372/181<br>60.7/59.9	Pbc[b] plus niraparib (300 mg) once daily in 28-day cycles until disease progression	Pbc[b] plus placebo until disease progression	PFS, grade ≥3 AEs, TDT
Coleman et al 2017]	ARIEL3 NCT01968213	III, multiple	375/189<br>61/62	Pbc[b] plus oral rucaparib 600 mg twice daily in 28 days cycles until disease progression	Pbc[b] plus placebo in 28 days cycles until disease progression	PFS, grade ≥3 AEs, TDT
Ledermann et al 2012	NCT00753545	II, multiple	131/123<br>58/59	Pbc[b] plus olaparib 400 mg twice daily until disease progression	Pbc[b] plus matching placebo until disease progression	PFS, OS, grade ≥3 AEs, TDT
Pujade-Lauraine et al 2017]	SOLO2 NCT01874353	III, multiple	286/295<br>56/56	Pbc[b] plus olaparib 300 mg twice daily until disease progression	Pbc[b] plus matching placebo until disease progression	PFS, grade ≥3 Aes
Oza et al 2015	NCT01081951	II, multiple	23/29<br>59/62	Olaparib (200 mg twice daily on day 1-10 every 21 days) plus Pacl (175 mg/m^2) plus Carb (AUC=4 on day 1 every 21 days) (4-6 cycles) plus olaparib (400 mg twice daily)	Pacl (175 mg/m^2) plus Carb (AUC=4 on day 1 every 21 days) (4-6 cycles)	PFS, OS, grade ≥3 AEs, TDT
Li et al 2021	NCT03863860	III, multiple	167/85<br>NA/NA	Pbc[b] plus fuzuloparib (150 mg, twice daily)	Pbc[b] plus placebo	PFS
Ledermann et al 2016	ICON6 NCT01968213	III, multiple	164/118<br>62/62	Pbc (every 3 wks) plus cediranib 20 mg once-daily (6 cycles) plus maintenance cediranib 20 mg once-daily	Pbc (every 3 wks) (6 cycles) plus maintenance placebo	PFS, OS, AEs, TDT
Pignata et	MITO16b	III,	203/203	Bev (10 mg/kg every 14	Carb (AUC=5) plus Pacl	PFS, OS,

al 2021	NCT01802749	multiple	60/61	days) plus PLD-Carb, or Bev (15 mg/kg every 21 days) plus Gemc-Carb or Pacl-Carb	(175 mg/m^2) every 21 days; Carb (AUC=4) plus Gemc (1,000 mg/m^2 on days 1 and 8) every 21 days; Carb (AUC=5) plus PLD (30 mg/m^2) every 28 days	grade ≥3 AEs
Mirza et al 2019	ENGOT-ov 24 NCT02354131	II, multiple	48/49<br>67/66	Bev (15 mg/kg 15 mg/kg every 21 days) plus niraparib (300 mg, once-daily)	niraparib (300 mg, once-daily)	PFS, AEs
Shi et al 2021	SOC-1 NCT01611766	III, multiple	182/175<br>55/53	secondary cytoreduction plus chemotherapy (10–14 days after surgery) (Pacl (175 mg/m^2) or docetaxel (75 mg/m^2) plus carbo (AUC=5) every 3 wks (6-9 cycles)); permitted of Bev or Parpi	chemotherapy (Pacl (175 mg/m^2) or docetaxel (75 mg/m^2) plus carbo (AUC=5) every 3 wks (6-9 cycles))	PFS, OS
Harter et al 2021	DESKTOP III NCT01166737	III, multiple	206/201<br>61/62	secondary cytoreduction plus Pbc.	Pbc alone.	PFS, OS

Appendix Table S2. Baseline characteristics of included studies. PFS=progression-free, OS=overall survival, ORR=objective response rate, AE=adverse events, TDT=treatment-discontinue for toxicity, NG=not given, AUC=area under the concentration-time curve, wks=weeks, Bev=bevacizumab, Carb=carboplatin, Gemc=gemcitabine, Pacl=paclitaxel, PLD=pegylated liposomal doxorubicin, Cisp=cisplatin, Trab=trabectedin, Pbc=platinum-based chemotherapy (carb-Pacl, Carb-Gemc, Carb-PLD, Carb, Cisp or nedaplatin), Parpi=poly (ADP-ribose) polymerase (PARP) inhibitors.

[a]Information of outcomes is incomplete, data cannot be extracted.

[b]Patients are treated with their last dose of platinum-based therapy within 8 weeks before receiving Parpi.

Nodes	Direct effect	Indirect effect	network	P
**Progression-survival**				
Carb-Paclfree vs Carb/Cis	-0.15 (-0.41 to 0.05)	-0.23 (-0.64 to 0.11)	-0.16 (-0.37 to 0.03)	0.64
Carb-Gemc vs Carb/Cis	-0.14 (-0.45 to 0.16)	-0.05 (-0.46 to0.38)	-0.12 (-0.31 to 0.11)	0.61
Carb-PLD vs Carb/Cis	-0.23 (-0.58 to 0.13)	-0.22 (-0.48 to 0.04)	-0.22(-0.41 to 0.03)	0.98
Carb-PLD vs Carb-Pacl	-0.04 (-0.23 to 0.24)	-0.11 (-0.45 to 0.29)	-0.06 (-0.20 to 0.15)	0.65

Carb-PLD vs Carb-Gemc	-0.16 (-0.50 to 0.18)	-0.06 (-0.46 to 0.35)	-0.11 (-0.34 to 0.11)	0.62
**Overall survival**				
Carb-Pacl vs Carb/Cis	-0.23 (-0.75 to 0.19)	-0.21 (-1.10 to 0.60)	-0.21 (-0.62 to 0.10)	0.97
Carb-Gemc vs Carb/Cis	-0.02 (-0.63 to 0.60)	-0.31 (-1.20 to 0.45)	-0.10 (-0.58 to 0.31)	0.46
Carb-PLD vs Carb/Cis	-0.37 (-1.00 to 0.27)	-0.13 (-0.70 to 0.40)	-0.20 (-0.65 to 0.15)	0.48
Carb-PLD vs Carb-Pacl	0.00 (-0.65 to 0.64)	0.01 (-0.71 to 0.78)	0.00 (-0.40 to 0.42)	0.97
Carb-PLD vs Carb-Gemc	0.02 (-0.62 to 0.65)	-0.27 (-1.10 to 0.47)	-0.10 (-0.57 to 0.34)	0.45
**Response rate**				
Carb-Pacl vs Carb/Cis	0.68 (0.06 to 1.41)	0.99 (-0.01 to 2.03)	0.77 (0.27 to 1.33)	0.59
Carb-Gemc vs Carb/Cis	0.70 (-0.12 to 1.52)	0.65 (-0.63 to 1.97)	0.70 (0.08 to 1.30)	0.93
Carb-PLD vs Carb/Cis	0.99 (-0.22 to 2.29)	0.57 (-0.04 to 1.28)	0.67 (0.14 to 1.26)	0.50
Carb-PLD vs Carb-Pacl	-0.14 (-0.61 to 0.32)	0.16 (-0.99 to 1.28)	-0.10 (-0.51 to 0.29)	0.62
Carb-PLD vs Carb-Gemc	0.06 (-1.07 to 1.14)	-0.06 (-1.04 to 1.10)	-0.02 (-0.68 to 0.70)	0.89

Appendix Table S3. Node-splitting analysis of inconsistency. P values are more than 0.05 indicating consistency between the direct effect and indirect effects. Carb=carboplatin, Cis=cisplatin, Pacl=paclitaxel, Bev=bevacizumab, Gemc=gemcitabine, PLD=pegylated liposomal doxorubicin, Trab=trabectedin, Zibo= zibotentan

a. PFS in initial therapy

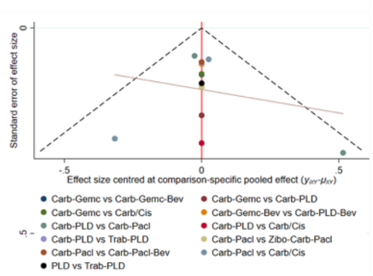

Egger's test			
Std_Eff	Coef.	Std. Err.	P>|t|
slope	-.3237487	.1830819	0.105
bias	.7184709	1.701385	0.681

b. OS in initial therapy

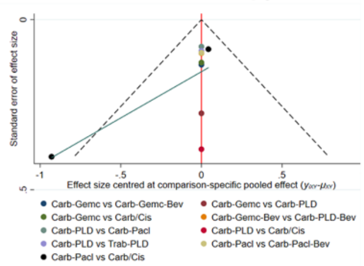

Egger's test			
Std_Eff	Coef.	Std. Err.	P>|t|
slope	.049273	.1078096	0.660
bias	-1.667305	.9422108	0.115

c. ORR in initial therapy

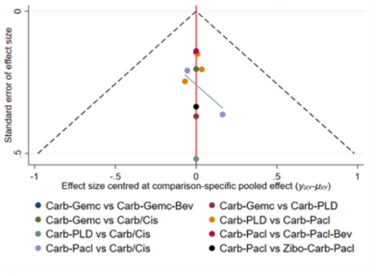

Egger's test			
Std_Eff	Coef.	Std. Err.	P>|t|
slope	.249684	.1997838	0.243
bias	-.4547891	.9688469	0.650

d. PFS in maintenance therapy

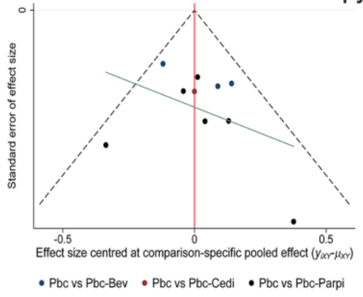

Egger's test			
Std_Eff	Coef.	Std. Err.	P>|t|
slope	.6307504	.5683834	0.304
bias	.9338093	1.506327	0.62

e. OS in maintenance therapy

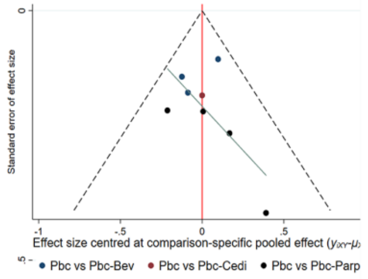

Egger's test			
Std_Eff	Coef.	Std. Err.	P>|t|
slope	-.1021721	.1572022	0.540
bias	-.0251762	.9729764	0.980

f. Grade ≥3 AEs in maintenance

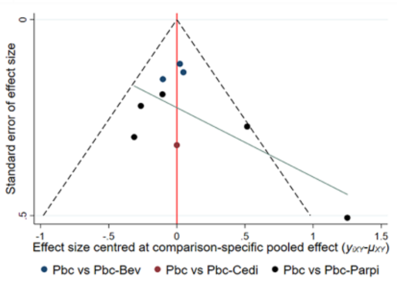

Egger's test			
Std_Eff	Coef.	Std. Err.	P>|t|
slope	-.2245164	.3941359	0.587
bias	3.92008	2.130837	0.108

g. AEs related discontinuation in maintenance therapy

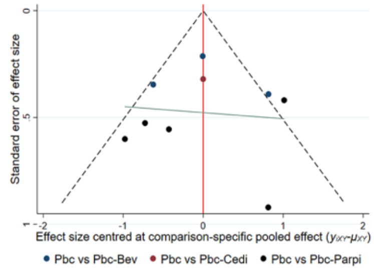

Egger's test			
Std_Eff	Coef.	Std. Err.	P>|t|
slope	-.311754	.2217372	0.193
bias	-3.871909	1.713038	0.052

Appendix Figure S1. Funnel plot and Egger regression test for publication bias**.** Carb=carbplatinum, Cis=cisplatin, Pacl=paclitaxel, Bev=bevacizumab, Gemc=gemcitabine, PLD=pegylated liposomal doxorubicin, Trab=trabectedin, Zibo=zibotentan, Pbc=platinum-based chemotherapy,

Bev=bevacizumab, Cedi=cediranib, Parpi=poly (ADP-ribose) polymerase (PARP) inhibitors, PFS=progression-free survival, OS=overall survival, ORR=objective response rate, AE=adverse events. Publication bias in Egger's test: P <0.05.

A

Comparison	Nausea and vomiting	Fatigue	Alopecia	Thrombocytopenia grade ≥3	Anemia grade ≥3	Allergy/immunology	Diarrhea	Neuropathy-Sensory
	**VS Carb or Cis**							
**Carb-PLD-Bev**	0.52	0.36	NA	0.38	0.53	NA	0.38	NA
**Carb-Pacl**	1.14	1.16	**0.05**	2.22	0.71	0.69	1.13	**0.24**
**Carb-Pacl-Bev**	1.01	0.90	**0.04**	4.13	0.48	0.42	0.76	0.30
**Carb-gemc-Bev**	0.64	0.43	NA	0.18	0.71	NA	0.51	NA
**Gemc-Carb**	0.89	0.65	0.33	0.23	1.07	0.77	0.78	0.96
**Carb-PLD**	0.84	1.31	0.67	0.36	0.82	3.58	0.38	1.00
**Zibo-Carb-Pacl**	1.12	1.60	0.04	0.10	1.07	NA	1.11	0.33
	**VS Carb-PLD-Bev**							
**Carb-Pacl**	2.17	3.22	NA	5.59	1.31	NA	2.96	NA
**Carb-Pacl-Bev**	1.94	2.51	NA	11.01	0.89	NA	1.96	NA
**Carb-gemc-Bev**	1.23	1.20	NA	0.47	1.32	NA	1.30	NA
**Gemc-Carb**	1.72	1.80	NA	0.60	1.98	NA	2.00	NA
**PLD-Carb**	1.60	3.61	NA	0.98	1.50	NA	0.99	NA
**Zibo-Carb-Pacl**	2.35	4.56	NA	0.84	2.3	NA	2.42	NA
	**VS Carb-Pacl**							
**Carb-Pacl-Bev**	0.90	0.78	0.87	1.94	0.68	0.61	0.69	1.27
**Carb-gemc-Bev**	0.57	0.37	NA	0.08	1.00	NA	0.44	NA
**Gemc-Carb**	0.79	0.56	**7.11**	0.11	1.53	1.12	0.68	**4.13**
**Carb-PLD**	0.74	1.12	**14.35**	**0.17**	1.15	5.03	0.34	**4.25**
**Zibo-Carb-Pacl**	0.94	1.57	0.94	0.65	1.37	NA	1.41	1.41
	**VS Carb-Pacl-Bev**							
**Carb-Gemc-Bev**	0.63	0.48	NA	0.04	1.45	NA	0.65	NA
**Gemc-Carb**	0.88	0.72	8.10	0.05	2.24	1.83	1.02	3.23
**Carb-PLD**	0.82	1.45	**16.53**	0.09	1.68	8.04	0.49	3.34
**Zibo-Carb-Pacl**	1.04	2.01	1.06	1.08	2.00	NA	2.08	1.11
	**VS Carb-Gemc-Bev**							
**Carb-Gemc**	1.39	1.49	NA	1.28	1.50	NA	1.54	NA
**Carb-PLD**	1.30	3.00	NA	2.09	1.13	NA	0.75	NA
**Zibo-Carb-Pacl**	1.92	3.75	NA	1.75	1.7	NA	1.81	NA
	**VS Carb-Gemc**							
**Carb-PLD**	0.94	2.01	2.01	1.59	0.76	4.57	0.48	1.03
**Zibo-Carb-Pacl**	1.4	2.51	0.13	1.83	1.14	NA	1.18	0.35

	VS PLD-Carb							
**Zibo-Carb-Pacl**	1.72	1.24	**0.07**	0.75	1.68	NA	1.76	0.36

B

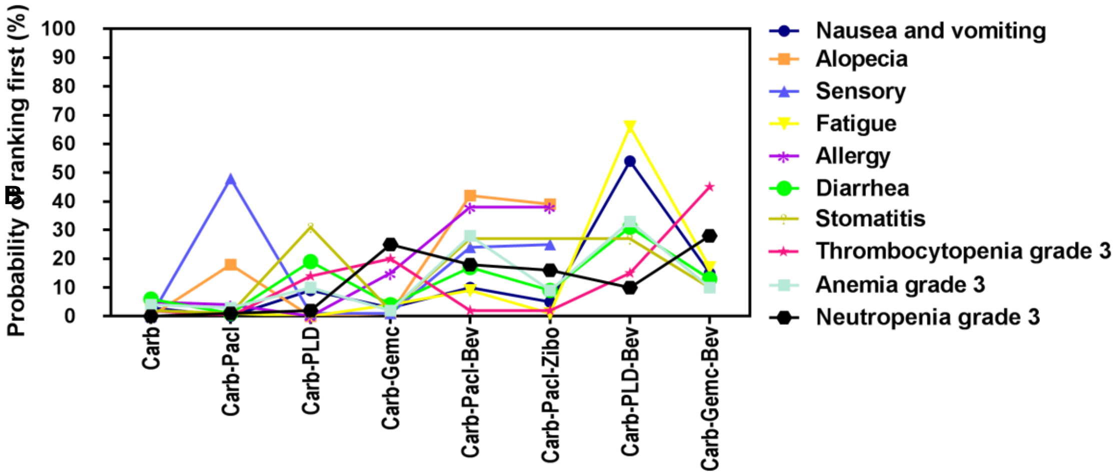

Appendix Figure S2. Ten kinds of commonly relative adverse events. **(A)** Pooled odds ratios for the specific adverse event Significant values are in bold and colored in blue (less toxicity) and light yellow (more toxicity) in a, **(B)** Ranking curves indicating the probability of ranking first among interventions. Carb=carboplatin, Pacl=paclitaxel, Bev=bevacizumab, Gemc=gemcitabine, PLD=pegylated liposomal doxorubicin, Trab=trabectedin, Zibo= zibotentan.

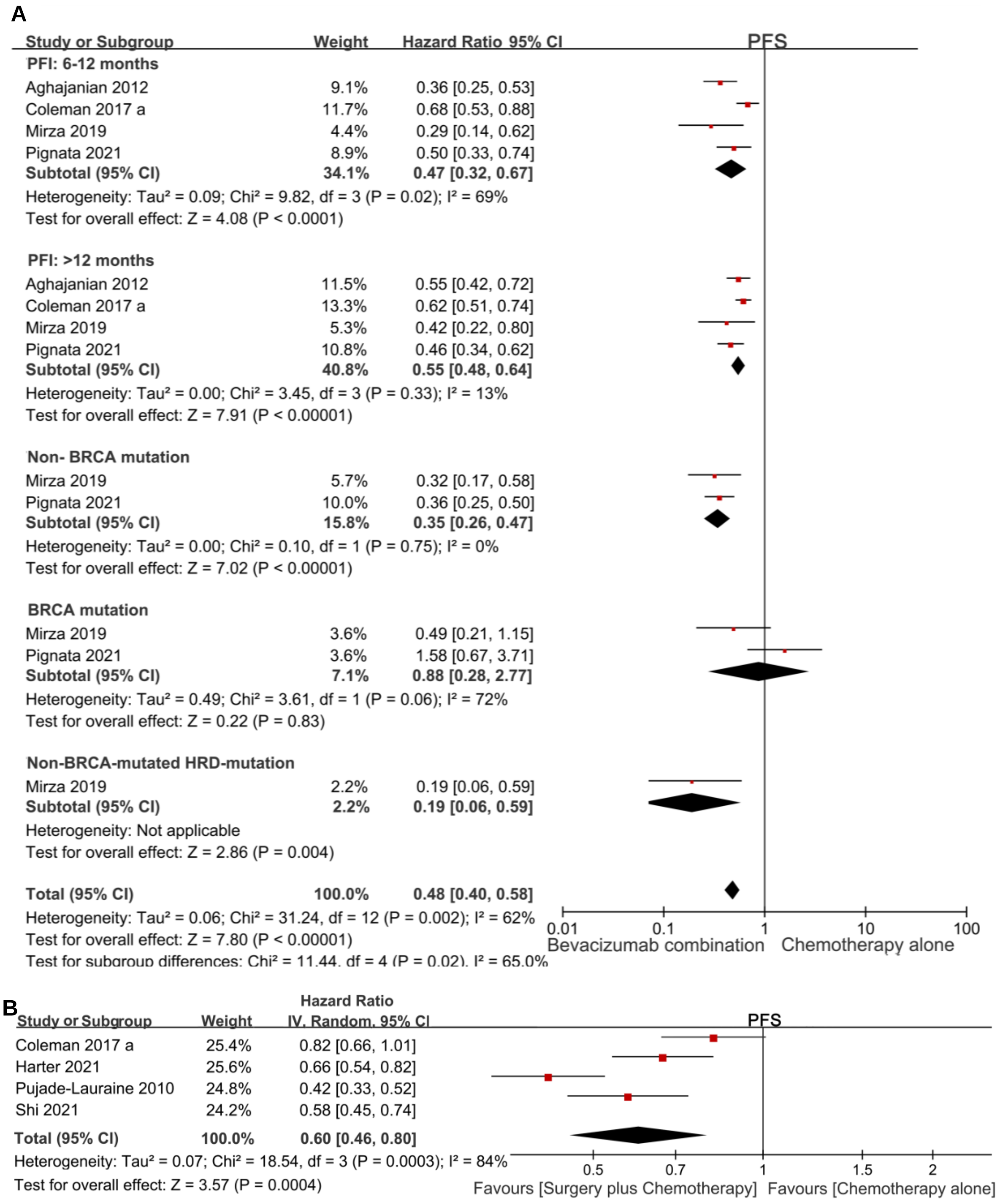

Appendix Figure S3. Forest plots of subgroup analysis of PFS in patients with PSROC. (A) Hazard ratios and 95% CIs for PFS in bevacizumab combination. (B) Hazard ratios and 95% CIs for secondary cytoreductive surgery plus chemotherapy. Hazard ratios of individual studies are shown by squares: the size of the square is the weight of the study; the horizontal line with the square represents the 95% confidence interval. The diamonds are the estimated pooled effect, random effect model. Evaluation of overall effect based on z-test. Abbreviations: PFS=progression-free survival, PFI= platinum-free interval, HRD= homologous recombination deficient.

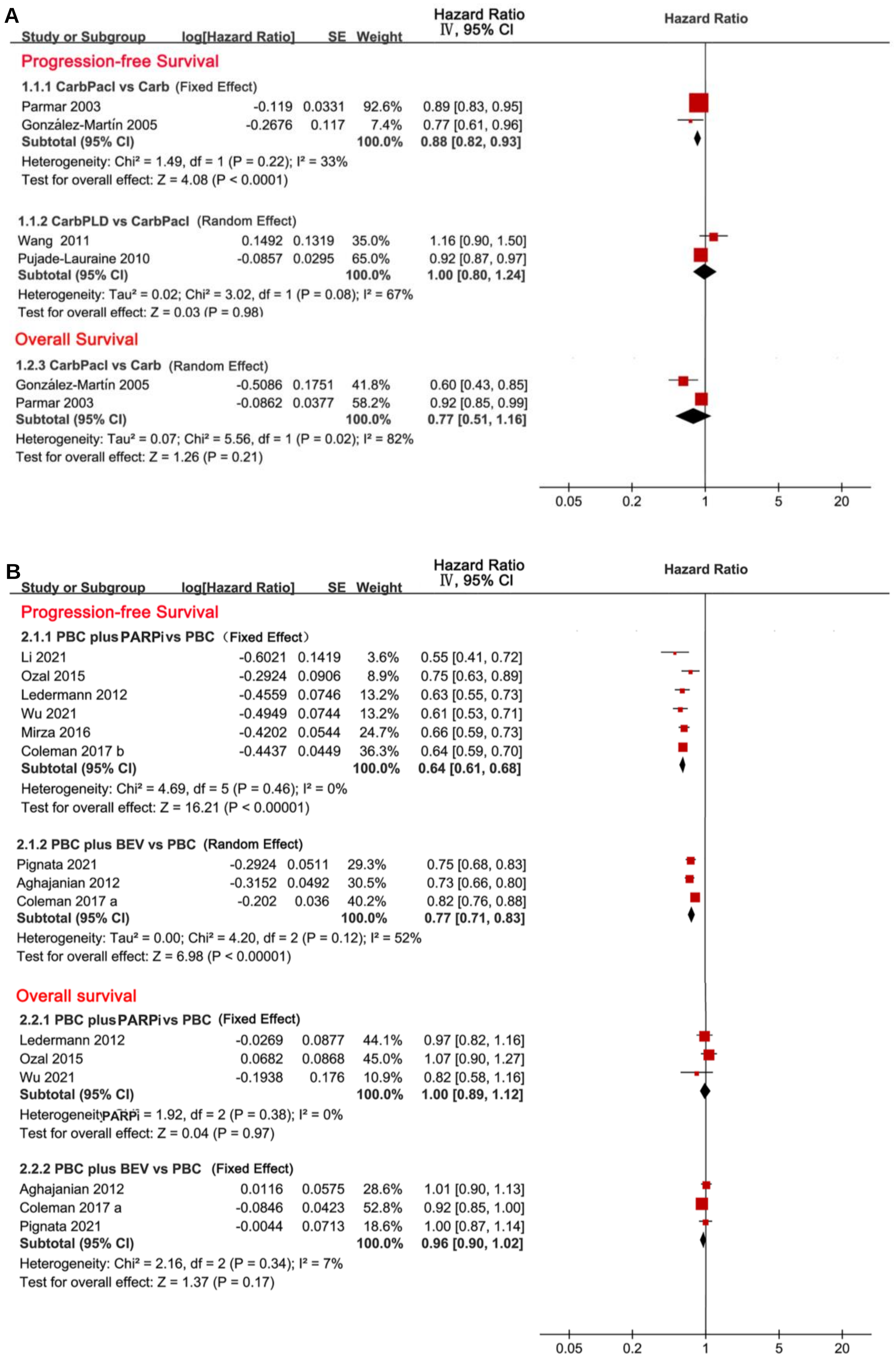
A
Study or Subgroup log[Hazard Ratio] SE Weight
Hazard Ratio IV, 95% CI
Hazard Ratio
Progression-free Survival
1.1.1 CarbPacl vs Carb (Fixed Effect)
Parmar 2003 -0.119 0.0331 92.6% 0.89 [0.83, 0.95]
González-Martín 2005 -0.2676 0.117 7.4% 0.77 [0.61, 0.96]
Subtotal (95% CI) 100.0% 0.88 [0.82, 0.93]
Heterogeneity: Chi² = 1.49, df = 1 (P = 0.22); I² = 33%
Test for overall effect: Z = 4.08 (P < 0.0001)
1.1.2 CarbPLD vs CarbPacl (Random Effect)
Wang 2011 0.1492 0.1319 35.0% 1.16 [0.90, 1.50]
Pujade-Lauraine 2010 -0.0857 0.0295 65.0% 0.92 [0.87, 0.97]
Subtotal (95% CI) 100.0% 1.00 [0.80, 1.24]
Heterogeneity: Tau² = 0.02; Chi² = 3.02, df = 1 (P = 0.08); I² = 67%
Test for overall effect: Z = 0.03 (P = 0.98)
Overall Survival
1.2.3 CarbPacl vs Carb (Random Effect)
González-Martín 2005 -0.5086 0.1751 41.8% 0.60 [0.43, 0.85]
Parmar 2003 -0.0862 0.0377 58.2% 0.92 [0.85, 0.99]
Subtotal (95% CI) 100.0% 0.77 [0.51, 1.16]
Heterogeneity: Tau² = 0.07; Chi² = 5.56, df = 1 (P = 0.02); I² = 82%
Test for overall effect: Z = 1.26 (P = 0.21)
0.05 0.2 1 5 20
B
Study or Subgroup log[Hazard Ratio] SE Weight
Hazard Ratio IV, 95% CI
Hazard Ratio
Progression-free Survival
2.1.1 PBC plus PARPi vs PBC（Fixed Effect）
Li 2021 -0.6021 0.1419 3.6% 0.55 [0.41, 0.72]
Ozal 2015 -0.2924 0.0906 8.9% 0.75 [0.63, 0.89]
Ledermann 2012 -0.4559 0.0746 13.2% 0.63 [0.55, 0.73]
Wu 2021 -0.4949 0.0744 13.2% 0.61 [0.53, 0.71]
Mirza 2016 -0.4202 0.0544 24.7% 0.66 [0.59, 0.73]
Coleman 2017 b -0.4437 0.0449 36.3% 0.64 [0.59, 0.70]
Subtotal (95% CI) 100.0% 0.64 [0.61, 0.68]
Heterogeneity: Chi² = 4.69, df = 5 (P = 0.46); I² = 0%
Test for overall effect: Z = 16.21 (P < 0.00001)
2.1.2 PBC plus BEV vs PBC (Random Effect)
Pignata 2021 -0.2924 0.0511 29.3% 0.75 [0.68, 0.83]
Aghajanian 2012 -0.3152 0.0492 30.5% 0.73 [0.66, 0.80]
Coleman 2017 a -0.202 0.036 40.2% 0.82 [0.76, 0.88]
Subtotal (95% CI) 100.0% 0.77 [0.71, 0.83]
Heterogeneity: Tau² = 0.00; Chi² = 4.20, df = 2 (P = 0.12); I² = 52%
Test for overall effect: Z = 6.98 (P < 0.00001)
Overall survival
2.2.1 PBC plus PARPi vs PBC (Fixed Effect)
Ledermann 2012 -0.0269 0.0877 44.1% 0.97 [0.82, 1.16]
Ozal 2015 0.0682 0.0868 45.0% 1.07 [0.90, 1.27]
Wu 2021 -0.1938 0.176 10.9% 0.82 [0.58, 1.16]
Subtotal (95% CI) 100.0% 1.00 [0.89, 1.12]
HeterogeneitPARPi = 1.92, df = 2 (P = 0.38); I² = 0%
Test for overall effect: Z = 0.04 (P = 0.97)
2.2.2 PBC plus BEV vs PBC (Fixed Effect)
Aghajanian 2012 0.0116 0.0575 28.6% 1.01 [0.90, 1.13]
Coleman 2017 a -0.0846 0.0423 52.8% 0.92 [0.85, 1.00]
Pignata 2021 -0.0044 0.0713 18.6% 1.00 [0.87, 1.14]
Subtotal (95% CI) 100.0% 0.96 [0.90, 1.02]
Heterogeneity: Chi² = 2.16, df = 2 (P = 0.34); I² = 7%
Test for overall effect: Z = 1.37 (P = 0.17)
0.05 0.2 1 5 20

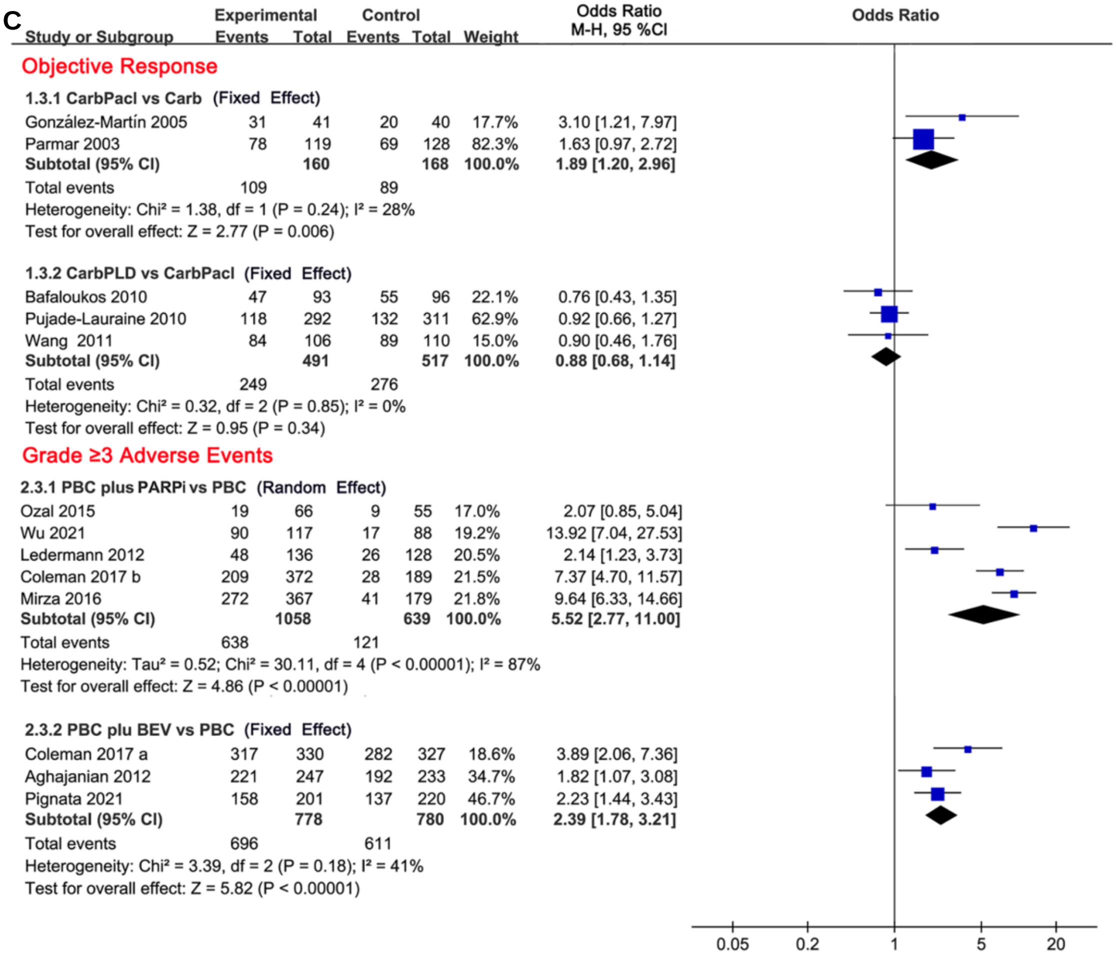

Appendix Figure S4. Forest plots describe the results of head-to-head comparisons. **(A)** Hazard ratios of progression-free survival and overall survival for patients receiving primary and maintain treatments, **(B)** Hazard ratios of progression-free survival and overall survival for patients receiving maintain treatments, **(C)** Odds ratio for objective response and grade ≥3 adverse events. Comparisons with one study were not plotted. Carb=carboplatin, Pacl=paclitaxel, Bev=bevacizumab, PLD=pegylated liposomal doxorubicin, PBC=platinum-based chemotherapy, BEV=bevacizumab, PARPi=poly (ADP-ribose) polymerase (PARP) inhibitors
